# The Antinociceptive Effect of Light-Emitting Diode Irradiation on Incised Wounds Is Correlated with Changes in Cyclooxygenase 2 Activity, Prostaglandin E2, and Proinflammatory Cytokines

**DOI:** 10.1155/2017/4792489

**Published:** 2017-04-02

**Authors:** Yuan-Yi Chia, Chien-Cheng Liu, Guan-Ming Feng, Chia-Chih Alex Tseng, Kuo-Chuan Hung, Chih-Chieh Chen, Ping-Heng Tan

**Affiliations:** ^1^Department of Anesthesiology, Kaohsiung Veterans General Hospital, Kaohsiung, Taiwan; ^2^Department of Anesthesiology, E-Da Hospital, I-Shou University, Kaohsiung, Taiwan; ^3^Department of Biological Sciences, National Sun Yat-sen University, Kaohsiung, Taiwan; ^4^Department of Plastic & Reconstructive Surgery, E-Da Hospital, I-Shou University, Kaohsiung, Taiwan; ^5^Department of Anesthesiology, National Cheng Kung University Medical College and Hospital, Tainan, Taiwan; ^6^School of Medicine, I-Shou University, Kaohsiung, Taiwan

## Abstract

*Background*. Light-emitting diode (LED) phototherapy has been reported to relieve pain and enhance tissue repair through several mechanisms. However, the analgesic effect of LED on incised wounds has never been examined.* Objectives*. We examined the analgesic effect of LED therapy on incision pain and the changes in cyclooxygenase 2 (COX-2), prostaglandin E2 (PGE2), and the proinflammatory cytokines interleukin 6 (IL-6), IL-1*β*, and tumor necrosis factor *α* (TNF-*α*).* Methods*. Rats received LED therapy on incised skin 6 days before incision (L-I group) or 6 days after incision (I-L group) or from 3 days before incision to 3 days after incision (L-I-L group). Behavioral tests and analysis of skin tissue were performed after LED therapy.* Results*. LED therapy attenuated the decrease in thermal withdrawal latency in all the irradiated groups and the decrease in the mechanical withdrawal threshold in the L-I group only. The expression levels of COX-2, PGE2, and IL-6 were significantly decreased in the three LED-treated groups, whereas IL-1*β* and TNF-*α* were significantly decreased only in the L-I group compared with their levels in the I groups (*p* < 0.05).* Conclusions*. LED therapy provides an analgesic effect and modifies the expression of COX-2, PGE2, and proinflammatory cytokines in incised skin.

## 1. Introduction

Low-intensity light therapy, commonly referred to as photobiomodulation, uses light in the far-red to near-infrared region (NIR) of the spectrum (630–1000 nm) and modulates numerous cellular functions. The light-emitting diode (LED), a light source, is as efficient as laser light and can be applied more economically [1]. Many advantageous effects of LED light treatment have been reported, such as increases in ATP synthesis [2], angiogenesis [3], collagen synthesis, fibroblast proliferation [2–4], inhibition of pain and oxidative stress [5, 6], and decreased inflammation [7]. Through several pathways, LED phototherapy can also diminish pain sensation and enhance tissue repair [4, 8].

Surgical trauma produces injuries in skin, fascia, muscle, and the nerve fibers innervating these tissues and induces subsequent postoperative acute pain. Although many basic and clinical research studies have improved our understanding of the pathologic mechanisms, controlling postsurgical pain well remains a challenge for physicians. The behavioral characteristics of a postoperative pain model in rats produced by hind paw incision resemble postoperative hypersensitivity in humans [9]. The nature of hypersensitivity in this pain model is different from that in other sustained pain models, such as the neuropathic pain model [10].

Since LED therapy can reduce the inflammation response, enhance tissue repair, and relieve pain, this therapy is considered a suitable method for preventing and treating symptoms of tissue damage after trauma or surgery. Thus, the antinociceptive effect of 940 nm wavelength LED therapy on acute pain resulting from tissue injury in a rat incision model was examined and the mechanism of antinociception was explored in this study.

## 2. Methods

### 2.1. Animals

Male Sprague–Dawley rats (180–220 g) were used in this study and were purchased from the National Science Council (Taiwan) according to the guidelines for pain research [11] and the ARRIVE guidelines [12]. All animal protocols were approved by the Institutional Review Board of I-Shou University, Kaohsiung, Taiwan. Two rats were housed per cage. The cages were maintained at 23 ± 1°C with a 12-hour light/dark cycle (lights on at 09:00), and the rats were left in the experimental room for 24 hours for acclimatization before the test.

### 2.2. Experimental Groups

The rats were randomly assigned to the following groups. Rats received LED therapy 6 days before incision (preincision group; L-I group) or 6 days after incision (postincision group; I-L group) or from 3 days before incision to 3 days after incision (preincision plus postincision group; L-I-L). Three groups of control rats received only a skin incision (incision only groups; I-3 h, I-3 d, and I-6 d groups) and underwent behavioral tests and tissue dissection at 3 hours, 3 days, or 6 days after skin incision for comparison to the L-I, L-I-L, and I-L groups, respectively. Three groups of LED-treated rats received a sham incision and the same LED therapy procedure on the contralateral paw for 6 days and were also considered as control (C) groups. The baseline mechanical withdrawal threshold and baseline thermal withdrawal latency were tested in naïve rats before undergoing a skin incision or LED irradiation, that is, before the incision was made in the I-6 d and I-L groups and before LED irradiation in the other groups. The mechanical withdrawal threshold and thermal withdrawal latency were then tested 3 hours after incision in the L-I and I-3 h groups, 3 days after incision in the L-I-L and I-3 d groups, and 6 days after incision in the I-L and I-6 d groups. Skin tissues dissected from all groups were collected for mRNA and protein analyses (*n* = 6) after behavioral testing. The staff members who performed the behavioral tests and collected and analyzed the skin tissues were blinded to the animal groups.

### 2.3. Incision Wound and Behavioral Test

An incision wound was made under 2% isoflurane in oxygen as anesthesia. The procedure was based on a previous report [9]; the plantar surface of one hind paw was prepared with 10% povidone-iodine solution, draped, and incised with a scalpel from the heel to the base of the toes. The flexor tendon from the heel to the toes was elevated with small forceps. The incision was then sutured with 5-0 nylon.

Pain behavior was tested by measuring the mechanical withdrawal threshold for mechanical allodynia and the thermal withdrawal latency for thermal hyperalgesia. To test the mechanical withdrawal threshold, a series of von Frey filaments (0.4, 0.6, 1, 2, 4, 6, 8, and 15 g; Stoelting, Wood Dale, IL, USA) were tested perpendicular to the plantar surface for 5-6 s for each filament. The 50% paw withdrawal threshold was determined using Dixon's up-and-down method [13]. Each threshold value measurement was used to calculate the threshold to withdrawal with a 50% probability to testing with calibrated von Frey filaments. These thresholds were determined 1-2 mm distal to the incision into the foot pad. Thermal sensitivity was evaluated using Plantar Test Apparatus (Ugo Basile, Comerio, Italy), and the data were presented as the paw withdrawal latency. The hind paw with or without the incised wound was heated by a calibrated radiant heat source, and the time to paw withdrawal was recorded. The radiant heat intensity was adjusted so that the basal paw withdrawal latency before incision was 10–12 s, with a cut-off of 20 s to prevent tissue damage. Each hind paw was tested three times. These three measurements were averaged for each animal.

### 2.4. LED Therapy

The rats in the LED-treated groups received LED irradiation on the left hind paw at a 940 nm wavelength for a period of 30 min to administer 4 J/cm^2^ of energy density at a power output of 160 mW. The therapeutic procedure began before or after the incision according to the assigned groups and was repeated every 24 hours. The rats were held in the ventral decubitus position to receive LED irradiation. The LED source was kept 1 cm above the injury at a 90° angle. A power checker (13PEM001/J, Melles Griot, Didam, Netherlands) was used to check the optical output power of the light source before the beginning of the experimental procedure. The equipment was specially developed for this experiment at the Department of Electronic Engineering of I-Shou University, Taiwan. After LED therapy, the animals were kept in their cages and observed until recovery from anesthesia.

### 2.5. Quantitative Real-Time Polymerase Chain Reaction (qRT-PCR)

One microgram of total RNA was used for cDNA synthesis and qRT-PCR gene expression analysis. First, reverse transcription (RT) was performed using a High Capacity cDNA Reverse Transcription Kit according to the manufacturer's protocol (Applied Biosystems, Foster City, CA). The reaction mixtures were incubated at 25°C for 10 min, 37°C for 120 min, and then 85°C for 5 sec. The final cDNA products were stored at −20°C until use. Next, the cDNA products were amplified by qRT-PCR on a 7500 Real-Time PCR System (Applied Biosystems, California, USA) using the SYBR® Green PCR Master Mix (Applied Biosystems, California, USA). The following thermal cycling program was used: 50°C for 2 min and 95°C for 10 min, followed by 40 cycles at 95°C for 15 sec and at 60°C for 1 min. Experiments were performed in triplicate for each data point. The primers used for qRT-PCR are listed in Table 1. *β*-Actin was used as a reference gene. The mRNA amounts of the genes of interest and *β*-actin were calculated from the threshold cycle (Ct) number. The relative expression level of each sample was normalized to the *β*-actin expression as an endogenous RNA control. The ΔCt values of the samples were determined as the difference between the Ct of the sample mRNA and the reference gene. ΔΔCt was determined as the difference between the ΔCt of the control groups (C groups) and the ΔCt of the other LED-treated groups. Data were expressed as 2^−ΔΔCt^ to provide an estimate of the amount of sample mRNA present in the LED-treated groups relative to the control group.

### 2.6. Western Blot Analysis

Total protein from skin tissue was prepared by the addition 1 : 20 of T-PER Tissue Protein Extraction Reagent (PIERCE, Rockford, IL, USA) (25 mM bicine, 150 mM sodium chloride [pH 7.6]) containing protease inhibitors [100 mM 4-(2-aminoethyl) benzenesulfonyl fluoride hydrochloride, 80 mM aprotinin, crystalline, 5 mM bestatin, 1.5 mM E-64, protease inhibitor, 2 M leupeptin, and 1 mM pepstatin A]. The tissue was homogenized with a homogenizer. After being placed on ice for 30 min, the homogenate was centrifuged at 10,000*g* for 15 min at 48°C. Proteins (30 *μ*g per lane) were separated in a 10% sodium dodecyl sulfate- (SDS-) polyacrylamide gel and transferred onto a polyvinylidene difluoride membrane (Merck Millipore, Darmstadt, Germany). The membrane was blocked with blocking solution (5% skim milk in TBST [2.42 g/L Tris-HCl, 80 g/L NaCl, and 0.1% Tween 20, pH 7.6]) for 0.5 hours and was rinsed briefly in TBST. The membrane was incubated overnight at 4°C with rabbit anti-PGE2 polyclonal antibody (1 : 5,000) (Bioss Inc., Woburn, MA, USA). A mouse anti-actin monoclonal antibody (1 : 10,000) (Millipore, Bedford, MA, USA) was used as a control.

After being rinsed with washing buffer for 30 min, the membrane was incubated for 1.5 hours with a horseradish peroxidase-conjugated secondary antibody: goat anti-rabbit IgG-HRP (1 : 10,000) (Santa Cruz Biotechnology, Texas, USA). The membrane was washed in washing buffer for 30 min, and the antibodies were then revealed using an enhanced chemiluminescence (ECL) detection kit (Merck Millipore, Darmstadt, Germany). For densitometric analyses, the blots were scanned and quantified with Image-Pro Plus analysis software (Media Cybernetics, Silver Spring, MD, USA), and the results were expressed as the ratio of PGE2 immunoreactivity to *β*-actin immunoreactivity.

### 2.7. Statistical Analysis

The Kolmogorov–Smirnov test was performed to verify the dependent variables' normality of distribution. The comparison of the mechanical withdrawal threshold and thermal withdrawal latency after treatment and baseline value in each group was analyzed using the paired *t-*test. The qRT-PCR data for COX-2, IL-6, IL-1*β*, and TNF-*α* and the western blot data for PGE2 were analyzed by the Kruskal-Wallis test to determine differences among groups followed by the Mann–Whitney *U* test for intergroup differences. The data were expressed as the means and standard deviation. Differences were considered statistically significant at *p* < 0.05. The statistical analysis was performed with SPSS software (14.0; SPSS Inc., Chicago, IL, USA).

## 3. Results

**Figure 1:**
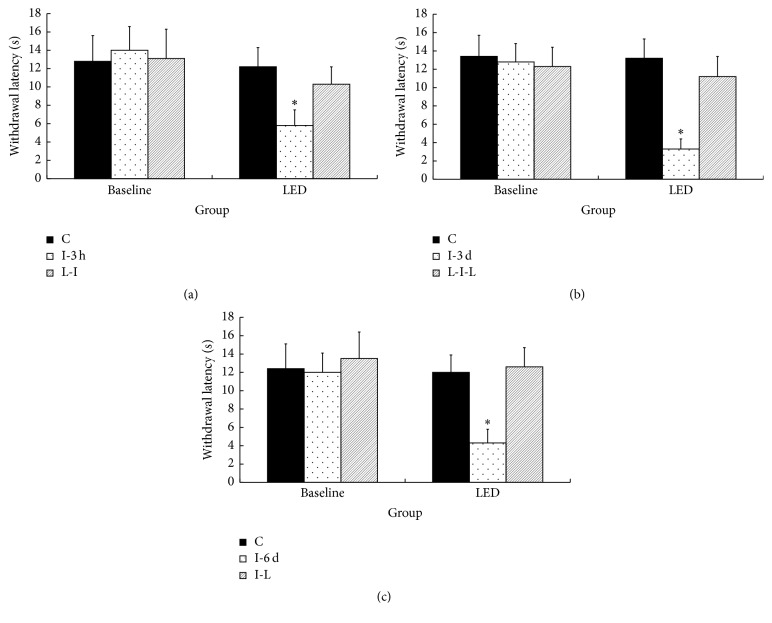
Mean withdrawal latency in the six groups (*n* = 6 each group) 6 days after LED irradiation performed before or after skin incision. C indicates LED irradiation for 6 days before or after sham skin incision on the paw contralateral to the LED-irradiated incised paw; I-3 h, I-3 d, and I-6 d indicate incision only groups that underwent behavioral testing 3 hours, 3 days, and 6 days after skin incision; L-I indicates LED irradiation for 6 days before skin incision; I-L indicates LED irradiation for 6 days after skin incision; L-I-L indicates LED irradiation from 3 days before skin incision to 3 days after skin incision. ^*∗*^*p* < 0.05 compared with the baseline value in each group. The values represent the means ± SD.

**Figure 2:**
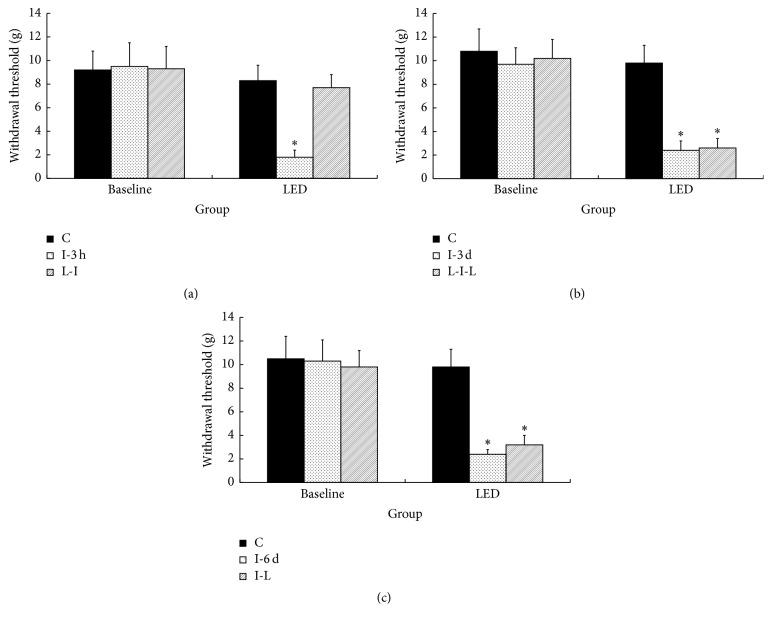
Mean withdrawal threshold in the six groups (*n* = 6 each group) 6 days after LED irradiation performed before or after skin incision. C indicates LED irradiation for 6 days before or after sham skin incision on the paw contralateral to the LED-irradiated incised paw; I-3 h, I-3 d, and I-6 d indicate incision only groups that underwent behavioral testing 3 hours, 3 days, and 6 days after skin incision; L-I indicates LED irradiation for 6 days before skin incision; I-L indicates LED irradiation for 6 days after skin incision; L-I-L indicates LED irradiation from 3 days before skin incision to 3 days after skin incision. ^*∗*^*p* < 0.05 compared with the baseline value in each group. The values represent the means ± SD.

The test of the analgesic effect of LED therapy on incision pain produced the following results. After the skin was incised, the thermal withdrawal latency was significantly decreased compared with the baseline value in three I groups (*p* < 0.05), but not in the three LED-treated groups (Figures 1(a), 1(b), and 1(c)). Significantly decreased mechanical withdrawal thresholds compared with the baseline value were noted after skin incision in the L-I-L and I-L groups but not in the L-I group (*p* < 0.05) (Figures 2(a), 2(b), and 2(c)).

**Figure 3:**
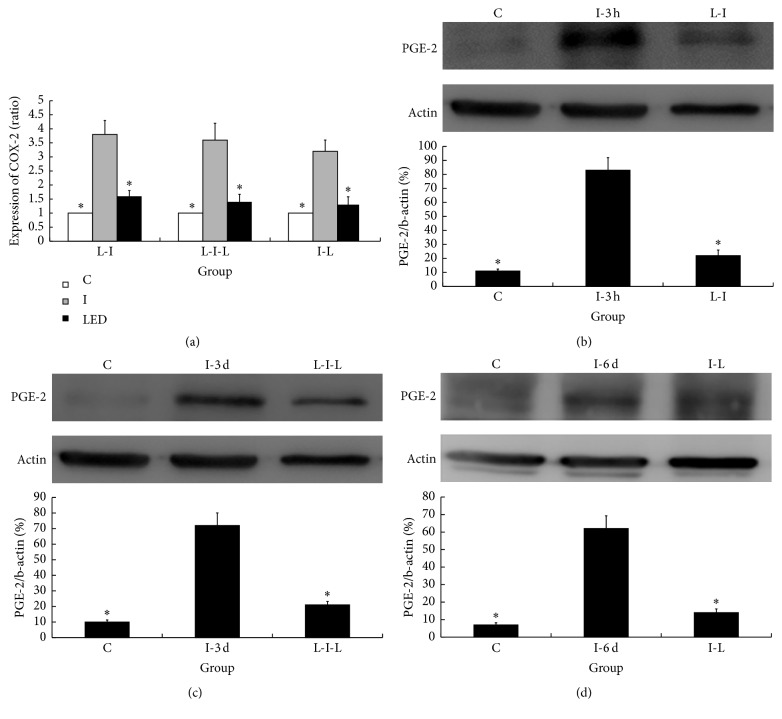
Changes in mRNA levels of cyclooxygenase-2 (COX-2) and representative western blots of prostaglandin E2 (PGE2) expression after LED irradiation. COX-2 mRNA is shown in (a), and representative western blots of PGE2 expression after LED irradiation are shown in (b, c, and d). Compared with the C groups, significant upregulation of COX-2 and PGE2 was noted in the I groups. Significantly decreased expression of COX-2 and PGE2 was noted in the LED irradiation groups compared with the incision only group. C indicates LED irradiation for 6 days on sham skin incision in the paw contralateral to the LED-irradiated paw; I-3 h, I-3 d, and I-6 d indicate incision only groups that underwent behavioral testing 3 hours, 3 days, and 6 days after skin incision; L-I indicates LED irradiation for 6 days before skin incision; I-L indicates LED irradiation for 6 days after skin incision; L-I-L indicates LED irradiation from 3 days before skin incision to 3 days after skin incision. ^*∗*^*p* < 0.05 compared with the I groups. The values represent the means ± SD.

**Figure 4:**
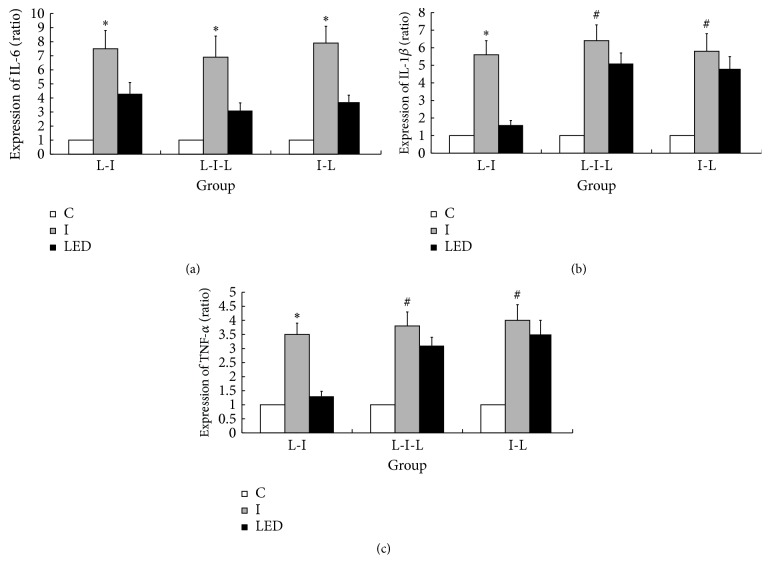
Changes in mRNA levels of interleukin-6 (IL-6), IL-1*β*, and tumor necrosis factor *α* (TNF-*α*) after LED irradiation. Compared with the C groups, significant upregulation of IL-1*β*, IL-6, and TNF-*α* was noted in all the I groups (a, b, and c). Significantly decreased expression of IL-6 was noted in all the LED-irradiated groups compared with the incision only group (a). After LED irradiation, significantly decreased expression of IL-1*β* and TNF-*α* was noted only in the L-I group (b, c). C indicates LED irradiation for 6 days on sham skin incision in the paw contralateral to the LED-irradiated paw; I indicates incision only; L-I indicates LED irradiation for 6 days before skin incision; I-L indicates LED irradiation for 6 days after skin incision; L-I-L indicates LED irradiation from 3 days before skin incision to 3 days after skin incision. ^*∗*^*p* < 0.05 compared with the C and LED-irradiated groups; ^#^*p* < 0.05 compared with the C groups. The values represent the means ± SD.

The production of IL-6, COX-2, PGE2, IL-1*β*, and TNF-*α* from skin tissue in response to incision and the inhibitory effects of LED irradiation are shown in Figures 3 and 4. Compared with the C groups, significant upregulation of COX-2, PGE2, IL-6, IL-1*β*, and TNF-*α* was noted. The expression levels of COX-2, PGE2, and IL-6 were significantly decreased in the LED-treated groups compared with those in groups I-3 h, I-3 d, and I-6 d (*p* < 0.05) (Figures 3(a), 3(b), 3(c), 3(d), and 4(a)). IL-1*β* and TNF-*α* were significantly decreased in the L-I group compared with their levels in group I-3 h (*p* < 0.05) (Figures 4(b) and 4(c)). However, compared with the I-6 d and I-3 d groups, in the I-L and L-I-L groups, no significant differences in the expression of IL-1*β* or TNF-*α* were noted (Figures 4(b) and 4(c)).

## 4. Discussion

In the present study, LED irradiation significantly alleviated thermal hyperalgesia in all three LED-treated groups and mechanical allodynia in only the L-I group. LED irradiation also significantly decreased the expression of IL-6, COX-2, and PGE2 in all the treatment groups and decreased IL-1*β* and TNF-*α* in the L-I group only. The measurement of cytokines was performed 3 hours, 3 days, or 6 days after incision in the L-I, L-I-L, and I-L groups, respectively. Thus, the inhibition of IL-1*β* and TNF-*α* by LED irradiation was short term and occurred only in the preemptive LED irradiation group (L-I group).

A previous study reported an increase in the level of IL-6, but not of IL-1*β* or TNF-*α*, in circulating blood 3 hours after carrageenan-induced inflammation. A prior injection of IL-6 antiserum abolished the induction of COX-2 activity and PGE2 release in vascular endothelial cells and attenuated thermal hyperalgesia [14, 15]. A-nociceptive inputs mediate mechanical secondary hyperalgesia [16]. Thermal latency reflects the activation of primarily C-fiber function [17]. Prostaglandins in the midbrain periaqueductal gray (PAG) matter exert tonic facilitatory control that targets C- rather than A-fiber-mediated spinal nociception [18]. Thermal hyperalgesia after infusion of PGE2 into the ventrolateral PAG has been observed [19]. Thus, the decrease in IL-6 observed in this study also contributed to the decreased expression of COX-2 and PGE2. The decrease in IL-6 and PGE2 also contributed to the attenuation of thermal hyperalgesia but not of mechanical allodynia.

The peripheral actions of cytokines in inflammatory pain include the activation of immune cells, such as mast cells, macrophages, and Schwann cells, to produce proinflammatory cytokines (e.g., IL-1*β*, TNF-*α*, and IL-6) [20]. These cytokines exert algesic effects directly by acting on nociceptors or indirectly through the release of other mediators, most notably prostanoids that are synthesized through COX-1 and COX-2 [21]. We showed that IL-6 concentrations were significantly decreased after LED irradiation, whereas TNF-*α* and IL-1*β* were not suppressed by LED irradiation in the I-L and L-I-L groups. A possible reason why TNF-*α* and IL-1*β* were not suppressed by LED irradiation in the I-L and L-I-L groups is that some amount of TNF-*α* and IL-1*β* was secreted by Schwann cells that were not inhibited by LED irradiation. LED irradiation was reported to be able to reduce the influx of inflammatory cells to the inflammation site [7]. However, whether LED irradiation could inhibit Schwann cells remains unclear. Another possible explanation is that the production of cytokines, such as TNF-*α* and IL-1*β*, is regulated at local inflammatory sites [22, 23], where their conversion to IL-6 was inhibited; thus, they remained increased in the tissue. IL-1*β* and TNF-*α* have also been reported to be associated with the development of allodynia and hyperalgesia [24, 25]; this could also be a reason why mechanical allodynia was not attenuated by LED irradiation in the L-I-L and I-L groups.

In addition to being blocked by IL-6, COX activity could be inhibited by LED irradiation. A study by Xavier et al. [26] showed that LED could inhibit the early chemotactic effects of inflammatory mediators by inhibiting COX. Similar results were observed by Campana et al. [27], who reported that LED therapy acted in the early stages of inflammation since a decrease in COX-2 mRNA was observed. Previous studies have shown that low-intensity LED could significantly reduce the mRNA expression of COX [28, 29], precursors such as phospholipase A2 (PLA2), and reactive oxygen species (ROS) [28]. In studies by Xavier and Lim [26, 28], irradiation at wavelengths of 880 nm and 635 nm was applied to Achilles tendinitis and human gingival fibroblasts. A wavelength of 940 nm was applied to damaged or sore muscles in studies by Vinck et al. [30] and Camargo et al. [31]. To the best of our knowledge, no researcher has evaluated the analgesic effect of LED irradiation on incised wounds. In this study, we applied 940 nm to incised wounds. The behavioral characteristics of a postoperative pain model in rats produced by hind paw incision resemble postoperative hypersensitivity in humans [9]. Our study was the first to apply 940 nm LED to reduce incision-induced nociception and the expression of COX-2, PGE2, and proinflammatory cytokines in incised wounds.

How does 940-nm light therapy inhibit the activation of COX enzymes and decrease the production of PGE2? In a previous study [28], light irradiation decreased the ROS level, indicating that irradiation functions as a ROS scavenger. The major effects of ROS during inflammation are the oxidative modification of PLA2 activation within the cell membrane and the stimulation of COX-2 mRNA expression [32, 33]. Therefore, light irradiation can directly disrupt ROS and subsequently inhibits the expression of cPLA2, sPLA2, and COX, resulting in the inhibition of PGE2 release.

Preemptive analgesia is an antinociceptive treatment administered before a noxious stimulus to prevent the establishment of neural nociception processing. Basic physiologic research has created great interest in the potential benefits of preemptive analgesia [34]. However, some clinical studies [35, 36] comparing the beneficial effects of preoperative analgesic treatments to the same treatment administered after incision did not show a significant preemptive effect. Our results showed that preemptive LED irradiation was effective at inhibiting the production of COX-2, PGE2, and IL-6. There was only a transient effect on the production of IL-1*β* and TNF-*α* 3 hours after the incision, but there was no effect on IL-1*β* or TNF-*α* more than three days after the incision. Similar results were reported by previous studies showing that the antinociceptive effects produced by nonsteroidal anti-inflammatory drugs (NSAIDs; COX inhibitors) were the same whether the NSAID was administered before or after the noxious stimulus [37, 38].

In conclusion, the results of the present study demonstrated that 940-nm LED phototherapy could effectively reduce incision-induced thermal hyperalgesia in all the LED-treated groups, regardless of whether irradiation occurred before or after the skin incision, whereas mechanical allodynia was reduced only 1 day after incision. The antithermal hyperalgesic and mechanical allodynic effects of this treatment are probably related to the decreased expression of COX-2, PGE2, IL-6, IL-1*β*, and TNF-*α*. Preemptive LED phototherapy transiently suppressed the expression of IL-1*β* and TNF-*α* several hours after incision. However, LED phototherapy could not suppress the expression of IL-1*β* or TNF-*α* beyond 3 days after incision. This LED therapy holds therapeutic potential for postsurgical pain.

## Figures and Tables

**Table 1:** Real-time polymerase chain reaction primers.

Gene	Direction	Primers
COX-2	Fwd.	5′-TGT ATG CTA CCA TCT GGC TTC GG-3′
	Rev.	5′-GTT TGG AAC AGT CGC TCG TCA TC-3′
IL-6	Fwd.	5′-CAT ATG TTC TCA GGG AGA TCT TGG A-3′
	Rev.	5′-CAG TGC ATC ATC GCT GTT CAT AC-3′
IL-1*β*	Fwd.	5′-CAC AGC AGC ATC TCG ACA AGA G-3′
	Rev.	5′-GAC ATA GGT AGC TGC CAC AGC TT-3′
TNF-*α*	Fwd.	5′-AGG CTG CCC CGA CTA TGT-3′
	Rev.	5′-AGG AGG CTG ACT TTC TCC-3′
*β*-Actin	Fwd.	5′-CGT ACC ACT GGC ATT GTG ATG-3′
	Rev.	5′-CAC GCT CGG TCA GGA TCT TC-3′

The COX-2, IL-6, IL-1*β*, TNF-*α*, and *β*-actin primer sequences were derived from the National Center for Biotechnology Information (Bethesda, Virginia) nucleotide sequence (accession numbers NM_017232.3, NM_012589.2, NM_031512.2, NM_012675.3, and NM_031144.3, resp.).
